# Association of GSTM1, GSTT1, and GSTP1 Gene Polymorphisms With Susceptibility to Periodontitis: A Systematic Review and Meta‐Analysis With Exploratory Analysis of Apical Periodontitis

**DOI:** 10.1155/ijod/4035228

**Published:** 2026-07-22

**Authors:** Imran Hossain, Md. Saifuddin Molla, Fatema-Tuz- Zohora

**Affiliations:** ^1^ Department of Pharmacy, University of Asia Pacific, 74/A Green Road Farmgate, Dhaka 1205, Bangladesh, uap-bd.edu; ^2^ Department of Clinical Pharmacy and Pharmacology, Faculty of Pharmacy, University of Dhaka, Dhaka 1000, Bangladesh, du.ac.bd; ^3^ Department of Statistics and Data Science, Jahangirnagar University, Savar, Dhaka 1342, Bangladesh, juniv.edu

**Keywords:** apical periodontitis, glutathione S-transferase, GSTM1, GSTP1, GSTT1, meta-analysis, periodontitis

## Abstract

**Background:**

Periodontitis is an inflammatory condition in which oxidative stress significantly contributes to tissue damage. Genetic polymorphisms in antioxidant enzymes, particularly glutathione S‐transferases (GSTs; GSTM1, GSTT1, and GSTP1), may influence an individual’s susceptibility to periodontal inflammatory conditions, including periodontitis and apical periodontitis (AP). However, prior studies have reported inconsistent results. Accordingly, this study evaluates the association between GST polymorphisms and periodontitis susceptibility, while also incorporating AP in an exploratory framework.

**Methods:**

Eight case–control studies were retrieved from PubMed, Scopus, and EBSCO. Pooled odds ratios (ORs) with 95% confidence intervals (CIs) were estimated using a random‐effects model due to high heterogeneity. Subgroup analyses were conducted by genotype, disease type, and ethnicity, along with exploratory pooled analyses combining periodontitis and AP. Study quality was evaluated using the Newcastle–Ottawa Scale (NOS).

**Results:**

Exploratory pooled analyses indicated that the GSTM1 null genotype was associated with increased risk (OR = 2.84, 95% CI: 1.07–7.56, *p*  = 0.04), whereas no significant association was identified in analyses restricted to periodontitis. In contrast, the GSTT1 null genotype and GSTP1 rs1695 polymorphism were not associated with periodontitis in either overall or subgroup analyses. The combined GSTM1/GSTT1 null genotype demonstrated a markedly elevated risk in the exploratory analysis (OR = 7.38, 95% CI: 1.69–32.19, *p*  = 0.008), but this association was not evident in the periodontitis‐only subgroup. Significant associations were primarily driven by the AP subgroup, while results for periodontitis alone were inconsistent.

**Conclusions:**

GSTM1 and GSTT1 polymorphisms may contribute to susceptibility to oral inflammatory conditions, particularly AP, whereas the evidence for periodontitis remains inconclusive. The findings highlight the potential role of compromised antioxidant defenses in disease pathogenesis. These results must be interpreted with caution as there was considerable heterogeneity and a small number of studies. Larger studies are required to validate these findings and elucidate possible mechanisms.

## 1. Introduction

Periodontitis is a chronic inflammatory condition driven by microbial biofilms and is a leading cause of tooth loss worldwide, involving the gradual breakdown of periodontal tissues [1]. According to the 2018 classification, periodontitis is now defined as a single disease entity based on staging and grading [2], although earlier studies used the terms chronic and aggressive periodontitis [3]. It is also among the most common oral complications worldwide, with ~12–13% of the population affected [4]. The condition gradually worsens the supportive periodontal framework, comprising the gingiva, the alveolar bone, and the periodontal ligament [5, 6]. Beyond its local effects, periodontitis has been increasingly recognized as a condition with systemic implications. Periodontal pathogens and their inflammatory responses can enter the bloodstream. This contributes to systemic inflammation and may influence diseases such as cardiovascular disorders and diabetes. These effects are mediated through shared mechanisms, including oxidative stress, immune dysregulation, and microbial dysbiosis [7].

However, while dental plaque bacteria are essential for disease initiation, host immune responses play a central role in tissue destruction, largely mediated through oxidative stress pathways [8]. Further, genetic variation is a significant factor in individual susceptibility to periodontitis, and there is growing evidence that polymorphisms of numerous genes are linked to disease risk [9–11]. Recent studies have further emphasized the role of cytokine gene polymorphisms, including interleukin‐related variants such as IL‐6 and IL‐10, in modulating host inflammatory responses and influencing susceptibility and severity of periodontitis [12, 13]. Moreover, periodontitis is increasingly recognized as a contributor to systemic inflammation and has been associated with conditions such as chronic kidney disease. These associations are mediated through shared mechanisms, including oxidative stress, immune dysregulation, and elevated proinflammatory cytokines. Importantly, genetic polymorphisms may influence this oral–systemic interaction, as demonstrated by studies linking inflammatory gene variants with both periodontal and systemic diseases [14].

In addition to periodontitis, apical periodontitis (AP; also referred to as periapical periodontitis) is another prevalent inflammatory condition of oral tissues, characterized by a host immune response to microbial infection localized at the root apex [15]. Elevated levels of oxidative stress markers, including 8‐hydroxydeoxyguanosine and oxidized glutathione, have been detected in AP samples [16], consistent with earlier evidence demonstrating disrupted redox homeostasis in this condition [17, 18]. Collectively, these findings reinforce the pivotal role of oxidative stress in the pathogenesis of both periodontitis and AP.

Glutathione S‐transferases (GSTs) are key phase II detoxification enzymes that facilitate the neutralization of reactive compounds and protect cellular components from oxidative damage [19]. There is a significant genetic polymorphism in human cytosolic GSTs with seven major classes: Alpha, Pi, Mu, Sigma, Theta, Omega, and Zeta [20]. Three of them, namely, the Mu (*µ*), Pi (*π*), and Theta (*θ*) classes, have seen the greatest amount of study, with genetic variations in these genes being shown to be linked to predisposition to a number of inflammatory diseases [21]. GSTM1 is located on chromosome 1 (1p13.3), and GSTT1 is situated on chromosome 22 (22p11.23). Homozygous null polymorphism in these genes results in complete loss of enzymatic activity and can lead to elevated oxidative stress [22]. Consequently, individuals carrying the null alleles exhibit reduced detoxification capacity, rendering them more vulnerable to a range of diseases, such as type 2 diabetes mellitus (T2DM) [23], various malignancies [24, 25], and liver disorders [26]. The human GSTP1 gene is situated on chromosome 11 (11q13.2) and holds a common polymorphism in exon 5, rs1695 (A313G, Ile105Val) that has possible functional importance. This is an amino acid replacement in which isoleucine or valine is present in the electrophilic‐reacting site of the GSTP protein, which could cause minor changes in the *α*‐helical and/or superhelical conformation. These structural modifications can alter the architecture of the H‐site, resulting in different substrate‐binding capacities and catalytic functions among GSTP enzyme variants [27]. GSTs that are reported to be expressed in cases of chronic inflammatory conditions are also suggested as possible biomarkers of periodontitis. Varghese et al. [19] found that the amount of GST in the gingival tissue and in the gingival crevicular fluid of the periodontitis patients was markedly reduced compared to that of the healthy controls. Despite the research on GST gene polymorphisms in different diseases, including periodontitis and AP, the results are not conclusive, probably due to the small sample size and different methodologies and population differences among participants [19, 28–31]. Therefore, a systematic and quantitative synthesis of available evidence is necessary to better clarify these associations.

Meta‐analysis, which synthesizes findings from multiple studies addressing the same research question, provides a more robust and comprehensive evaluation by increasing statistical power and reducing random error. As a result, it often provides more reliable evidence than individual studies and has become increasingly important in genetic association research. Although periodontitis and AP are distinct clinical entities, the pulp and periodontal tissues function as a closely integrated biologic unit. These tissues share embryonic, anatomical, and functional interrelationships. Communication between the pulp and the periodontal tissue occurs through anatomical pathways, including the apical foramen, as well as lateral and accessory canals, and dentinal tubules. Such anatomical and microbiological interconnections facilitate the bidirectional spread of infection and inflammation between the pulp and periodontal tissues, contributing to the development of combined endodontic‐periodontal lesions [32]. These shared mechanisms suggest that genetic susceptibility factors, particularly polymorphisms in antioxidant enzymes such as GSTs, may influence both conditions. Accordingly, this study systematically reviews and quantitatively synthesizes evidence on the association between GST polymorphisms (GSTM1, GSTT1, and GSTP1) and periodontitis susceptibility, with exploratory consideration of AP. To ensure methodological rigor, these conditions were analyzed separately in subgroup analyses, while combined analyses were interpreted cautiously to provide a broader understanding of the role of GST polymorphisms in oral inflammatory diseases.

## 2. Materials and Methods

### 2.1. Database Search Strategy

A comprehensive search of PubMed, Scopus, and EBSCO databases was conducted to identify studies examining the association between GSTM1, GSTT1, and GSTP1 gene polymorphisms and susceptibility to periodontitis and AP up to January 2026; however, additional databases such as Web of Science, Embase, and the Cochrane Library were not searched, which may be a limitation. To minimize the risk of missing relevant studies, a comprehensive manual search of the reference lists of all retrieved articles was also conducted. The search strategy consisted of both Medical Subject Headings (MeSH) and free‐text keywords, including “GSTM1,” “GSTT1,” “GSTP1,” “glutathione S‐transferase,” “rs1695,” “Ile105Val,” “I105V,” “A313G,” “periodontitis (including terms used in earlier classifications such as ‘chronic’ and ‘aggressive’ periodontitis),” as well as related terms such as “apical periodontitis” and “periapical periodontitis” to capture studies investigating periapical inflammatory conditions. Although periodontitis and AP are distinct clinical entities, both involve inflammatory and oxidative stress‐related mechanisms; therefore, AP studies were included in exploratory analyses and analyzed separately. Additional polymorphism‐related terms such as “polymorphism,” “genotype,” “single nucleotide polymorphism (SNP),” “variant,” and “gene deletion” were considered during search development; however, these terms were not applied as mandatory filters to avoid excluding relevant genetic association studies that may not explicitly include such terminology in titles or abstracts. No language restrictions were applied, and only studies involving human subjects were considered.

### 2.2. Selection Criteria

To be included, studies had to satisfy the following criteria: (1) the studies were case–control studies that explored the relationship between GST gene polymorphisms (GSTM1, GSTT1, and GSTP1) and periodontal inflammatory conditions (periodontitis and AP); (2) the case group comprised patients with a clinical diagnosis of periodontitis (including chronic and aggressive forms based on earlier classifications) or AP, whereas the control group included periodontally healthy subjects; (3) they reported the relevant data, including odds ratios (ORs) with 95% confidence intervals (CIs) of the genotype or allele frequency, including sufficient raw data to compute them; and (4) in cases of multiple publications using overlapping datasets, only the study with the most complete dataset and/or largest sample size was included after careful comparison of study populations and recruitment periods. Studies were removed in the following criteria: (1) they were not genetic polymorphism studies; (2) genotype or allele frequency data were vague or incomplete; (3) the case sample had periodontitis and other systemic disease patients; (4) they were conference abstracts or other nonoriginal publications; and (5) no control group was included.

### 2.3. Data Extraction

All retrieved studies were screened by two independent investigators (Imran Hossain and Md. Saifuddin Molla) using their titles and abstracts, and records that failed to meet the eligibility criteria were excluded. In cases of disagreement, consensus was achieved through discussion or with input from a third reviewer (Fatema‐Tuz‐Zohora). Whole papers of the possibly relevant articles were subsequently accessed and evaluated. A standardized, piloted data extraction form was used to ensure consistency among reviewers. The data obtained involved the following information of every eligible study: first author, year of publication, country, ethnicity, total cases and controls, and genotype and allele frequencies of the GSTM1, GSTT1, and GSTP1 genes, as well as the type of periodontal condition (periodontitis or AP) for subgroup analysis. The Hardy–Weinberg equilibrium (HWE) *p* value was calculated for control groups in GSTP1 studies only, as HWE assessment was not applicable to deletion polymorphisms (GSTM1 and GSTT1). A *p*‐value < 0.05 was considered indicative of deviation from equilibrium. The quality of the selected studies was assessed using Newcastle–Ottawa Scale (NOS) [33]. Research with an NOS score of 6 or higher was considered high quality.

### 2.4. Statistical Analysis

The studies evaluating the associations between GST gene polymorphisms and periodontitis and AP were analyzed by computing pooled ORs with their respective 95% CIs. The statistical heterogeneity of the studies was assessed using the Cochran Q test and the *I* ^2^ statistic [34]. An *I* ^2^ value >50% was considered indicative of substantial heterogeneity. Pooled effect estimates were calculated using a random‐effects model to account for potential heterogeneity among studies [35, 36]. Observed heterogeneity was used in selecting the appropriate analytical model. Subgroup and sensitivity analyses were conducted based on disease type (periodontitis vs. AP) and ethnicity to explore potential sources of heterogeneity. All statistical analyses were conducted using Review Manager (RevMan) software, version 5.4 (The Cochrane Collaboration, Copenhagen, Denmark). The meta‐analysis was prepared and reported in accordance with the Preferred Reporting Items of a Systematic Review and a Meta‐Analysis (PRISMA 2020) guidelines [37]. This review was not registered in PROSPERO due to the exploratory nature of combining periodontitis and AP; however, all methods were defined a priori following PRISMA 2020 guidelines.

## 3. Results

### 3.1. Characteristics of Included Studies

**Figure 1:**
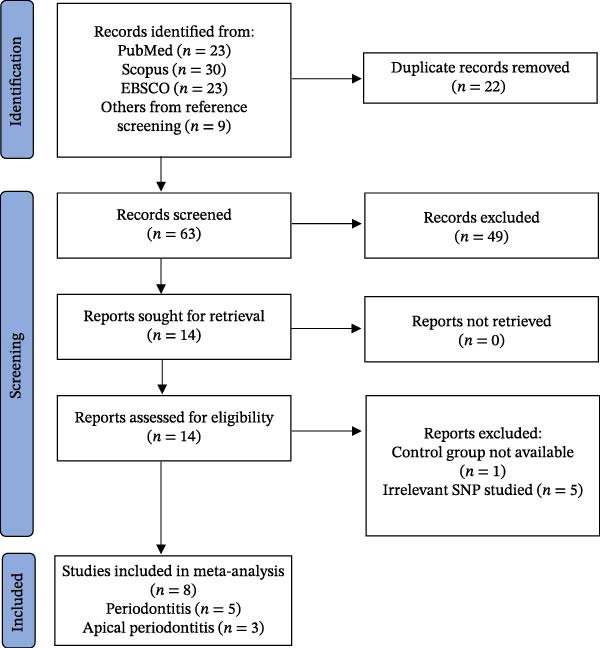
Flow diagram illustrating the literature screening and study selection process.

Eight case–control studies were analyzed, including 1124 diseased participants and 1206 healthy participants. Analyses were restricted to individuals with available genotype data (Figure 1). For GSTM1, six studies contributed to the exploratory overall analysis, including four on periodontitis and two on AP. For GSTT1, five studies were included: three on periodontitis and two on AP. For GSTP1 rs1695, four studies were included: three on periodontitis and one on AP. The overall pooled analyses were considered exploratory because they combined periodontitis and AP. Accordingly, subgroup analyses were conducted based on disease type and, where applicable, ethnicity. Table 1 summarizes the main features of these studies. The NOS scores of the included studies ranged from 7 to 9, indicating no studies with poor methodological quality. Thus, the information from these eight articles was combined and discussed in the current meta‐analysis.

**Table 1:** Overview of the studies included in this meta‐analysis.

First author	Year	Country/ethnicity	Cases/controls	Genotyping	Polymorphism	_*p*HWE_ in controls for GSTP1	NOS
Periodontitis							
Izakovicova et al. [29]	2024	Czech Republic/Caucasian	203/204 (200/150 for GSTP1)	Multiplex PCR; allele‐specific PCR	GSTM1; GSTT1; GSTP1	0.2636	8
Arshad et al. [28]	2023	Pakistan/South Asian	203/201	Multiplex PCR; T‐ARMS‐PCR	GSTM1; GSTT1; GSTP1	0.0010	7
Saravanan et al. [38]	2023	India/South Asian	50/50	PCR‐RFLP	GSTP1	0.1022	7
Concolino et al. [39]	2007	Italy/Caucasian	83/125	Multiplex PCR	GSTM1; GSTT1	—	9
Kim et al. [40]	2004	South Korea/East Asian	115/120	Multiplex PCR	GSTM1	—	7
Apical periodontitis							
Bagryantseva [41]	2025	Russia/Caucasian	150/50	Real‐time PCR	GSTP1	0.2840	8
Kadić et al. [31]	2025	Bosnia and Herzegovina and Serbia/Caucasian	200/250	Multiplex PCR	GSTM1; GSTT1	—	7
Jakovljevic et al. [30]	2020	Serbia/Caucasian	120/200	Multiplex PCR	GSTM1; GSTT1	—	7

*Note:*_*p*HWE_ values are reported only for studies evaluating GSTP1 polymorphism in control groups. A dash (–) indicates that _*p*HWE_ was not applicable (e.g., for GSTM1 and GSTT1 null polymorphisms).

Abbreviations: NOS, Newcastle‐Ottawa Scale; PCR, polymerase chain reaction; PCR‐RFLP, polymerase chain reaction‐restriction fragment length polymorphism; _*p*HWE_, *p* value in Hardy‐Weinberg equilibrium; T‐ARMS‐PCR, tetra‐primer amplification refractory mutation system PCR.

### 3.2. Impact of GST Gene Polymorphisms (GSTM1, GSTT1, and GSTP1) on Susceptibility to Periodontitis and AP

**Figure 2:**
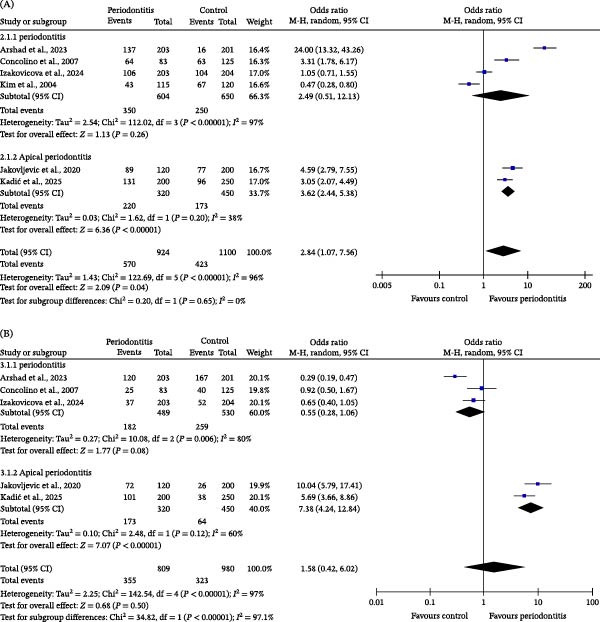
Forest plots presenting the relationship between GSTM1 (A) and GSTT1 (B) polymorphisms and susceptibility to periodontitis and apical periodontitis, including subgroup and exploratory pooled analyses. CI, confidence interval; GSTM1, glutathione S‐transferase mu 1; GSTT1, glutathione S‐transferase theta 1; OR, odds ratio.

A pooled estimate from six studies indicated that the GSTM1 null polymorphism was associated with increased risk (OR = 2.84, 95% CI: 1.07–7.56, *p* = 0.04; *I* ^2^ = 96%) (Table 2 and Figure 2A). However, substantial heterogeneity and a wide confidence interval suggest caution in interpretation. In the periodontitis‐only subgroup, analysis did not reveal a significant association involving the GSTM1 null polymorphism. No significant association was identified in ethnicity‐stratified analyses for either Caucasian or Asian populations. In contrast, in the AP subgroup, GSTM1 null polymorphism showed a significant association (OR = 3.62, 95% CI: 2.44–5.38, *p* < 0.00001; *I* ^2^ = 38%), with all studies conducted in Caucasian populations.

**Table 2:** Meta‐analysis of GSTM1, GSTT1, and GSTP1 polymorphisms and the risk of periodontitis and AP.

Groups	Disease subgroup	Ethnicity subgroup	Number of studies	Association test			Heterogeneity test	
				OR	95% CI	*p* value	*I* ^2^ (%)	*p* value
GSTM1 null	Overall (exploratory)	—	6	2.84	1.07–7.56	0.04	96	<0.00001
	Periodontitis	Overall	4	2.49	0.51–12.13	0.26	97	<0.00001
		Caucasian	2	1.82	0.59–5.60	0.30	89	0.002
		Asian	2	3.36	0.07–159.89	0.54	99	<0.00001
	Apical periodontitis	Overall	2	3.62	2.44–5.38	<0.00001	38	0.20
		Caucasian	2	3.62	2.44–5.38	<0.00001	38	0.20

GSTT1 null	Overall (exploratory)	—	5	1.58	0.42–6.02	0.50	97	<0.00001
	Periodontitis	Overall	3	0.55	0.28–1.06	0.08	80	0.006
		Caucasian	2	0.74	0.51–1.08	0.12	0	0.38
		Asian	1	0.29	0.19–0.47	<0.00001	NA	NA
	Apical periodontitis	Overall	2	7.38	4.24–12.84	<0.00001	60	0.12
		Caucasian	2	7.38	4.24–12.84	<0.00001	60	0.12

GSTM1/T1 null/null	Overall (exploratory)	—	5	7.38	1.69–32.19	0.008	94	<0.00001
	Periodontitis	Overall	3	2.47	0.64–9.56	0.19	92	<0.00001
		Caucasian	2	1.43	0.40–5.10	0.58	84	0.01
		Asian	1	7.03	3.80–13.01	<0.00001	NA	NA
	Apical periodontitis	Overall	2	41.70	19.98–87.02	<0.00001	0	0.74
		Caucasian	2	41.70	19.98–87.02	<0.00001	0	0.74

GSTM1/T1 either null	Overall (exploratory)	—	5	2.37	1.17–4.82	0.02	89	<0.00001
	Periodontitis	Overall	3	1.50	0.77–2.94	0.24	76	0.02
		Caucasian	2	1.27	0.53–3.05	0.59	78	0.03
		Asian	1	2.15	1.15–4.00	0.02	NA	NA
	Apical periodontitis	Overall	2	4.33	2.55–7.35	<0.00001	56	0.13
		Caucasian	2	4.33	2.55–7.35	<0.00001	56	0.13

GSTM1/T1 present/present	Overall (exploratory)	—	5	0.42	0.21–0.85	0.02	89	<0.00001
	Periodontitis	Overall	3	0.66	0.33–1.31	0.23	77	0.01
		Caucasian	2	0.78	0.38–1.88	0.59	78	0.03
		Asian	1	0.45	0.24–0.83	0.01	NA	NA
	Apical periodontitis	Overall	2	0.23	0.14–0.39	<0.00001	56	0.13
		Caucasian	2	0.23	0.14–0.39	<0.00001	56	0.13

GSTP1 allelic (G vs. A)	Overall (exploratory)	—	4	1.18	0.42–3.32	0.76	95	<0.00001
	Periodontitis	Overall	3	1.60	0.47–5.41	0.45	96	<0.00001
		Caucasian	1	0.94	0.67–1.31	0.71	NA	NA
		Asian	2	2.09	0.32–13.80	0.44	96	<0.00001
	Apical periodontitis	Overall	1	0.47	0.30–0.75	0.001	NA	NA
		Caucasian	1	0.47	0.30–0.75	0.001	NA	NA

GSTP1 homozygous (GG vs. AA)	Overall (exploratory)	—	4	1.20	0.25–5.70	0.82	90	<0.00001
	Periodontitis	Overall	3	1.97	0.36–10.88	0.43	87	0.0004
		Caucasian	1	1.16	0.48–2.78	0.74	NA	NA
		Asian	2	2.59	0.14–47.88	0.52	92	0.0004
	Apical periodontitis	Overall	1	0.29	0.12–0.69	0.005	NA	NA
		Caucasian	1	0.29	0.12–0.69	0.005	NA	NA

GSTP1 heterozygous (AG vs. AA)	Overall (exploratory)	—	4	1.15	0.42–3.17	0.78	90	<0.00001
	Periodontitis	Overall	3	1.55	0.48–5.02	0.47	92	<0.00001
		Caucasian	1	0.81	0.52–1.26	0.35	NA	NA
		Asian	2	2.19	0.49–9.81	0.31	88	0.003
	Apical periodontitis	Overall	1	0.45	0.21–1.00	0.05	NA	NA
		Caucasian	1	0.45	0.21–1.00	0.05	NA	NA

GSTP1 dominant (AG + GG vs. AA)	Overall (exploratory)	—	4	1.14	0.35–3.64	0.83	94	<0.00001
	Periodontitis	Overall	3	1.62	0.43–6.07	0.48	94	<0.00001
		Caucasian	1	0.85	0.56–1.30	0.46	NA	NA
		Asian	2	2.25	0.35–14.33	0.39	94	<0.00001
	Apical periodontitis	Overall	1	0.38	0.18–0.79	0.009	NA	NA
		Caucasian	1	0.38	0.18–0.79	0.009	NA	NA

GSTP1 recessive (GG vs. AG + AA)	Overall (exploratory)	—	4	1.26	0.36–4.44	0.72	86	<0.00001
	Periodontitis	Overall	3	1.84	0.42–8.02	0.42	84	0.002
		Caucasian	1	1.27	0.54–2.99	0.58	NA	NA
		Asian	2	2.22	0.16–30.36	0.55	91	0.0001
	Apical periodontitis	Overall	1	0.45	0.22–0.91	0.03	NA	NA
		Caucasian	1	0.45	0.22–0.91	0.03	NA	NA

*Note*: All pooled estimates were calculated using a random‐effects model. All studies included in the apical periodontitis subgroup were conducted in Caucasian populations. Subgroups containing a single study did not allow estimation of heterogeneity (*I* ^2^ and *p* value not applicable). Exploratory analyses include both periodontitis and apical periodontitis and should be interpreted cautiously.

Abbreviations: CI, confidence interval; *I* ^2^, heterogeneity statistic; NA, not applicable; OR, odds ratio.

No significant association was identified for GSTT1 in the overall exploratory analysis (Table 2 and Figure 2B). In the periodontitis subgroup, the association remained nonsignificant. Ethnicity‐stratified analysis showed no significant association in Caucasian populations. In contrast, a significant association was identified in Asian populations (OR = 0.29, 95% CI: 0.19–0.47, *p* < 0.00001). A strong association was observed in the AP subgroup, in which the GSTT1 null genotype was associated with increased risk (OR = 7.38, 95% CI: 4.24–12.84, *p* < 0.00001; *I* ^2^ = 60%).

**Figure 3:**
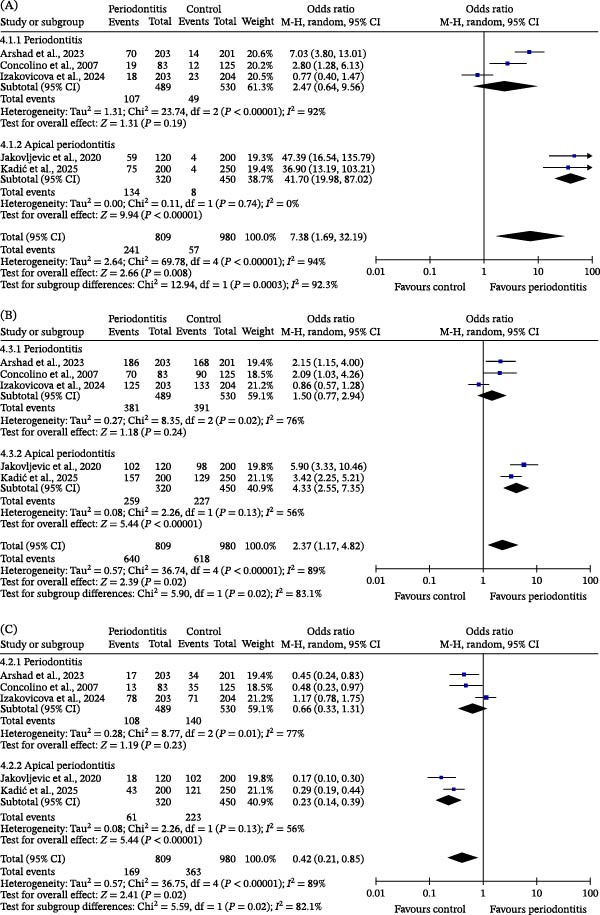
Forest plots depicting the relationship between combined GSTM1/GSTT1 genotypes and susceptibility to periodontitis and apical periodontitis, with results presented for subgroup and exploratory pooled analyses: (A) null/null, (B) either‐null, and (C) present/present polymorphisms. GSTM1, glutathione S‐transferase mu 1; GSTT1, glutathione S‐transferase theta 1; 95% CI, 95% confidence interval; OR, odds ratio.

Evaluation of combined genotypes demonstrated a significant association for the GSTM1/GSTT1 double null genotype in the exploratory model (OR = 7.38, 95% CI: 1.69–32.19, *p* = 0.008; *I* ^2^ = 94%) (Figure 3A and Table 2). No significant association was identified in the periodontitis subgroup. In contrast, a markedly increased risk was observed in AP (OR = 41.70, 95% CI: 19.98–87.02, *p* < 0.00001; *I* ^2^ = 0%). The either‐null genotype was also associated with increased risk in the exploratory analysis (OR = 2.37, 95% CI: 1.17–4.82, *p* = 0.02; *I* ^2^ = 89%). This association was not significant in the periodontitis subgroup, whereas a significant effect was evident in AP (OR = 4.33, 95% CI: 2.55–7.35, *p* < 0.00001) (Figure 3B and Table 2). A protective effect was observed for the present/present genotype in the exploratory analysis (OR = 0.42, 95% CI: 0.21–0.85, *p* = 0.02; *I* ^2^ = 89%). No significant association was detected in the periodontitis subgroup. A significant protective effect was again evident in AP (OR = 0.23, 95% CI: 0.14–0.39, *p* < 0.00001) (Figure 3C and Table 2).

**Figure 4:**
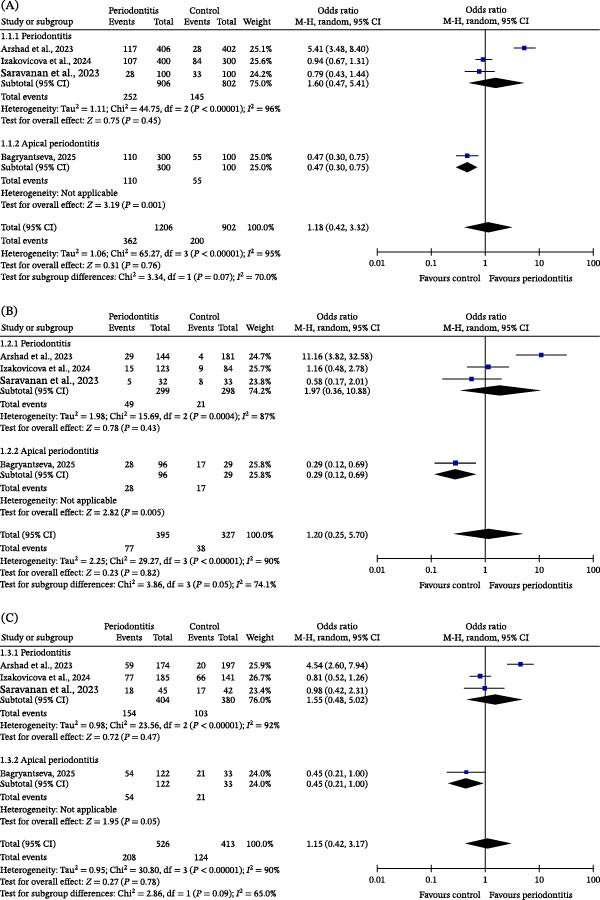
Forest plots summarizing the association of GSTP1 rs1695 (A313G) polymorphism with susceptibility to periodontitis and apical periodontitis across subgroup and exploratory pooled analyses under allelic (A), homozygous (B), and heterozygous (C) models. GSTP1, glutathione S‐transferase Pi 1; 95% CI, 95% confidence interval; OR, odds ratio.

**Figure 5:**
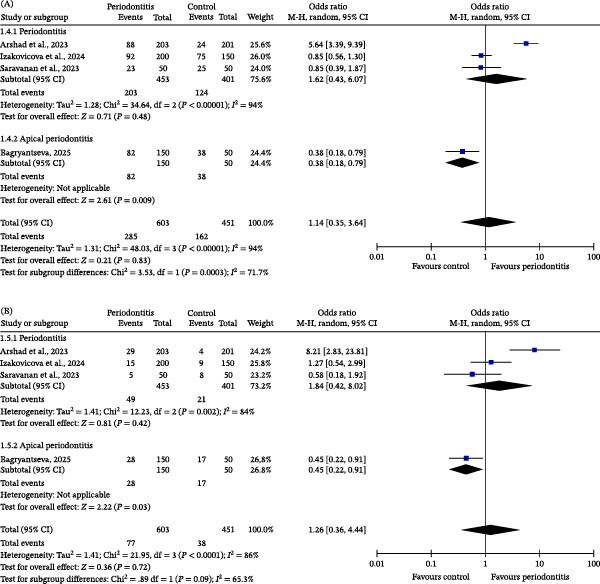
Forest plots depicting the relationship between GSTP1 rs1695 (A313G) polymorphism and susceptibility to periodontitis and apical periodontitis, with results presented for subgroup and exploratory pooled analyses under dominant (A) and recessive (B) models. GSTP1, glutathione S‐transferase Pi 1; 95% CI, 95% confidence interval; OR, odds ratio.

Analysis of GSTP1 rs1695 (A313G) did not indicate a significant association with periodontitis across any genetic model, including allelic (OR = 1.18, 95% CI: 0.42–3.32, *p* = 0.76; *I* ^2^ = 95%), homozygous (OR = 1.20, 95% CI: 0.25–5.70, *p* = 0.82), heterozygous (OR = 1.15, 95% CI: 0.42–3.17, *p* = 0.78), dominant (OR = 1.14, 95% CI: 0.35–3.64, *p* = 0.83), and recessive models (OR = 1.26, 95% CI: 0.36–4.44, *p* = 0.72) (Figures 4 and 5). Subgroup analyses showed no significant association between periodontitis and ethnicity. In contrast, significant associations were observed in AP within Caucasian populations, particularly under the allelic (OR = 0.47, 95% CI: 0.30–0.75, *p* = 0.001) and homozygous models (OR = 0.29, 95% CI: 0.12–0.69, *p* = 0.005).

Significant associations observed in exploratory analyses were largely driven by the AP subgroup.

### 3.3. Sensitivity Analysis

Sensitivity analysis using a leave‐one‐out approach revealed that, for the GSTT1 null polymorphism in the periodontitis subgroup, removal of Arshad et al. [28] eliminated heterogeneity (80% to 0%) without altering the nonsignificant association (*p* = 0.08 to 0.12), and the overall exploratory analysis remained stable.

In contrast, for combined GSTM1/GSTT1 genotypes, the results were sensitive to the inclusion of Izakovicova et al. [29]. Removal of this study reduced heterogeneity and changed the periodontitis subgroup results from nonsignificant to significant for the null/null (OR: 2.47–4.58), either‐null (OR: 1.50–2.13), and present/present genotypes (OR: 0.66–0.46). The overall exploratory analyses were also affected, with increased effect sizes for null/null (OR: 7.38–12.99) and either‐null genotypes (OR: 2.37–3.18) and a stronger protective effect for the present/present genotype (OR: 0.42–0.31), indicating limited robustness and substantial influence of this study.

For the GSTP1 rs1695 polymorphism, the removal of Arshad et al. [28] markedly reduced heterogeneity across all genetic models in both the periodontitis subgroup and overall exploratory analysis but did not alter statistical significance. The pooled estimates shifted toward a protective trend. Notably, this study deviated from HWE, which may partly explain its influence on heterogeneity.

### 3.4. Publication Bias Analysis

Because fewer than 10 studies were included in each analysis, formal statistical tests for publication bias (e.g., Egger’s test) were not performed, as these methods are underpowered and potentially misleading in small samples. In line with current recommendations, funnel plots were not used to infer publication bias, and no conclusions regarding publication bias were drawn. This limitation should be taken into account when interpreting the findings.

## 4. Discussion

Overall, the present meta‐analysis suggests that GSTM1 and GSTT1 polymorphisms may contribute to susceptibility to oral inflammatory conditions, with stronger and more consistent associations observed in AP compared to periodontitis alone.

Periodontitis is a common chronic inflammatory condition caused by imbalances in oral biofilms, which disrupt the natural oral microbiome and gradually damage the periodontal ligament, cementum, and alveolar bone, creating deep periodontal pockets and often leading to tooth loss [42]. GSTs are important in counteracting the effects of lipid peroxidation products, including hydroperoxides, during oxidative stress in periodontal inflammation. They also participate in the decontamination of xenobiotics, especially polychlorinated aromatic hydrocarbons and hydroxylated derivatives of benzo‐*α*‐pyrene [43–45]. These are consistent with the broader role of oxidative stress‐related biomarkers in the progression of periodontitis. Alterations in antioxidant defense mechanisms, including GST enzyme activity, may influence the balance between oxidative damage and tissue repair, thereby contributing to disease severity and progression [46, 47]. Similarly, recent studies have highlighted that cytokine‐related genetic polymorphisms, including variants in interleukin‐6 and interleukin‐10, play critical roles in modulating host inflammatory responses and influencing susceptibility and severity of periodontitis [12, 13].

Over the last few years, the genetic variability of GST enzymes has been slowly gaining value as a possible risk factor for periodontitis. Kim et al. [40] were the first to document a higher risk of periodontitis in individuals with the GSTM1 (+) genotype in a Korean population of 115 periodontitis patients and 126 healthy controls (OR = 2.1 and 95% CI = 1.3–3.6). Analysis stratified by smoking status revealed that the GSTM1(+) carriers were severely affected with a high risk of periodontitis among the smokers (OR = 3.1) and moderately affected with a moderate risk of the disease among nonsmokerss (OR = 1.8). Conversely, Concolino et al. [39] found a significant link between periodontitis and the GSTM1‐null genotype in an Italian population (OR = 3.59) without any references to smoking status, age, sex, and oral hygiene behavior. However, no statistically significant differences were observed in the distribution of the GSTT1 genotype. Moreover, Ortega et al. [48] examined the GSTM1, GSTT1, and GSTP1 polymorphisms in a pilot study with 60 periodontitis patients (30 smokers and 30 nonsmokers) in Mexico (no control group; hence not included in the present meta‐analysis). While no differences in the GSTT1 and GSTP1 polymorphisms were observed among smokers and nonsmokers, smokers with periodontitis were more likely to have the GSTM1(+) genotype (*p* ≤ 0.05). It is interesting to note that the frequencies of null polymorphisms in GSTM1 and GSTT1 and the mutant GSTP1 genotype were higher in participants with periodontitis than in historical data from a healthy Mexican population. Recently, in a case–control study with 200 AP patients and 250 healthy controls, Kadić et al. [31] found that the null genotypes of GSTM1 and GSTT1 and the case of the combination of GSTM1 and GSTT1 were significantly linked with the increased risk of AP. The findings are generally in line with previous research by Arshad et al. [28], who evaluated 203 periodontitis patients and 201 controls and found that the absence of GSTM1, together with the presence of GSTT1, was associated with an elevated risk of periodontitis. In addition, carriers of the mutant G allele of the GSTP1 (rs1695) polymorphism are found to have significantly higher susceptibility to periodontitis in the same study. Nevertheless, these findings do not align with those of Izakovicova et al. [29]. Even though they analyzed 203 periodontitis participants and 204 healthy controls, their study did not show any significant differences in the distribution of GSTM1 or GSTT1 genotypes between cases and controls. Furthermore, no significant link was observed between the GSTP1 rs1695 polymorphism and periodontitis susceptibility in that population. In general, the findings of research studies investigating the relationship between periodontitis and GST gene polymorphisms have been inconsistent and contradictory. The majority of included studies were conducted in Caucasian populations from European regions, which enhances the applicability of findings within these groups; however, variability across ethnic backgrounds remains an important source of heterogeneity in genetic association studies. Differences in allele frequencies and genetic backgrounds across populations, along with variations in inclusion and exclusion criteria and analytical approaches, may partly explain the inconsistent findings observed among studies [49]. In addition, variability in treatment approaches and clinical management strategies may also contribute to inconsistencies across studies. Previous literature has highlighted that outcomes in periodontal and peri‐implant disease management are often heterogeneous due to differences in study design, treatment protocols, and diagnostic criteria, which can influence observed associations and limit comparability between studies [50]. Thus, to achieve a more accurate and detailed estimate of the association between GST gene polymorphisms (GSTM1, GSTT1, and GSTP1) and periodontitis susceptibility, we performed a meta‐analysis of 8 eligible case–control studies.

Among the limited number of pooled studies, this meta‐analysis provides an early systematic evaluation of the association between GST polymorphisms (GSTM1, GSTT1, and GSTP1) and periodontitis susceptibility, with exploratory consideration of AP. However, it is important to emphasize that the overall pooled analyses were exploratory combining studies on periodontitis and AP. The results demonstrate that the GSTT1/GSTM1 dual‐null, either null, and the GSTM1 null genotypes are significantly correlated with a higher risk of oral inflammatory conditions, primarily driven by the AP subgroup. Notably, this significant association was primarily driven by the AP subgroup, whereas no statistically significant associations were observed in the periodontitis‐only subgroup or in ethnicity‐based analyses. Conversely, the GSTT1/GSTM1 dual‐positive genotype was found to be protective. Similarly, these protective effects were not consistently observed in periodontitis‐only analyses. Taken together, these findings suggest that GSTM1 and GSTT1 polymorphisms may contribute to susceptibility to oral inflammatory diseases, but their role in periodontitis specifically remains inconclusive based on current evidence. However, despite biological plausibility and positive associations reported in some individual studies, no significant relationship was identified between the GSTP1 rs1695 (A313G) polymorphism and periodontitis susceptibility under any genetic model in pooled analysis.

Sensitivity analyses revealed that several findings, particularly for combined GSTM1/GSTT1 genotypes in the periodontitis subgroup, were not robust and were influenced by individual studies. Specifically, the removal of Izakovicova et al. [29] substantially altered both effect sizes and statistical significance, indicating instability in these associations. Similarly, Arshad et al. [28], which deviated from HWE, were identified as a major contributor to heterogeneity, especially in GSTP1 analyses. Although the exclusion of this study reduced heterogeneity across all genetic models, it did not materially change the overall nonsignificant findings for GSTP1, suggesting that the lack of association is relatively consistent. These observations highlight that the current evidence is sensitive to individual studies and should be interpreted with caution.

An important finding of the present study is the differential association observed between periodontitis and AP. While significant associations were consistently observed in the AP subgroup across multiple genetic models, such associations were not evident in periodontitis‐only analyses. This discrepancy may be attributed to differences in disease pathogenesis, sample size, or study design. However, both conditions share common underlying mechanisms, including oxidative stress, inflammatory responses, and host‐mediated tissue destruction, which may explain the stronger and more consistent genetic effects observed in apical lesions. These findings support the rationale for exploratory combined analysis while emphasizing the need for cautious interpretation when generalizing results to periodontitis alone.

It is important to note that differences in confounding variables may have contributed to between‐study heterogeneity. These include dietary patterns, socioeconomic status, maternal age, smoking, oral hygiene practices, systemic diseases, genetic factors, variation in diagnostic criteria, and access to healthcare. This is supported by emerging evidence that genetic polymorphisms in inflammatory pathways may influence both periodontal and systemic conditions, such as chronic kidney disease, highlighting broader oral‐systemic interactions [14]. In addition, the limited number of included studies and the combined analysis of periodontitis and AP may restrict the generalizability of the findings. The exploratory nature of combined analyses, the high heterogeneity across several models, and the sensitivity of results to individual studies further reduce the robustness of the conclusions. The high heterogeneity observed may be attributed to differences in ethnicity, study design, and disease classification; however, meta‐regression could not be performed due to the limited number of studies. The combined analysis was exploratory and should not be interpreted as evidence of a unified disease mechanism. The certainty of evidence was not formally assessed (e.g., using GRADE), which is also a limitation. Therefore, future well‐planned, high‐quality prospective studies are justified in order to better control the possible confounders and to further confirm the findings of this meta‐analysis.

## 5. Conclusion

This meta‐analysis suggests that genetic variations in GST genes may influence susceptibility to oral inflammatory diseases, particularly AP, while the evidence for periodontitis alone remains inconclusive. In exploratory pooled analyses, GSTM1 null, GSTM1/GSTT1 double null, and either null genotypes were associated with increased risk, whereas present/present genotype showed protective effects; however, these findings were largely driven by the AP subgroup and were not consistently observed in periodontitis‐only analyses. The GSTT1 null genotype and GSTP1 rs1695 polymorphism were not significantly associated overall or in periodontitis‐only analyses. These findings indicate that impaired GST‐mediated antioxidant defense may contribute to disease pathogenesis, particularly in apical lesions. However, given the limited number of studies, high heterogeneity, and sensitivity of results to individual studies, further large‐scale, well‐designed investigations are needed to confirm these associations and clarify their role in periodontitis.

## Author Contributions

**Imran Hossain**: conceptualization, methodology, data curation, formal analysis, software, visualization, statistical analysis, writing – original draft. **Md. Saifuddin Molla**: data curation, software, formal analysis, writing – review and editing. **Fatema-Tuz- Zohora**: supervision, validation, writing – review and editing.

## Funding

This study did not receive any specific funding.

## Disclosure

All authors have read and approved the final version of the manuscript. Fatema‐Tuz‐Zohora had full access to all data in this study and takes full responsibility for the integrity and accuracy of the data analysis.

## Ethics Statement

Ethical approval was not required for this study as it is a systematic review and meta‐analysis based on previously published data. No new data involving human participants or animals were collected, and all included studies were conducted in accordance with their respective ethical standards.

## Conflicts of Interest

The authors declare no conflicts of interest.

## Data Availability

The authors confirm that the data supporting the findings of this study are available within the article.
